# Household economic burden and catastrophic expenditures in non-resistant tuberculosis patients: cross-sectional survey in Guizhou, China

**DOI:** 10.3389/fpubh.2025.1510195

**Published:** 2025-05-30

**Authors:** Xiaoxue Ma, Aijue Huang, Huijuan Chen, Jian Zhou, Yuying He, Weibing Wang, Rong Du, Xueli Guo, Qi Zhao, Jinlan Li

**Affiliations:** ^1^Guizhou Provincial Center for Disease Control and Prevention, Guiyang, China; ^2^School of Public Health, Fudan University, Shanghai, China

**Keywords:** tuberculosis, economic burden, catastrophic expenditures, influence factor, economically disadvantaged families

## Abstract

**Objectives:**

In accordance with the World Health Organization (WHO)'s “End TB Strategy,” which aims to eradicate catastrophic expenditures faced by TB-affected families, we intend to thoroughly investigate and comprehend the economic burden, catastrophic expenditures, and contributing factors pertaining to non-drug-resistant tuberculosis patients’ families in Guizhou Province. Our goal is to formulate policy recommendations that can effectively alleviate the financial strain on these patients and their families.

**Methods:**

The pulmonary tuberculosis cases, which were non-drug-resistant, registered across the province during May–June 2020, and successfully treated at the time of the survey, underwent questionnaire interviews conducted through probability proportional sampling. Utilizing the WHO methodology, the household economic burden borne by these patients was computed, with the mean and median (interquartile range), abbreviated as “M (IQR),” employed to describe the economic burden, and the proportion (%) used to depict catastrophic expenditures. Further analysis of the factors influencing catastrophic expenditures within these families was conducted using chi-squared (*χ*^2^) tests and binary logistic regression.

**Results:**

The average total out-of-pocket expenses (OOP) incurred by 2,283 non-drug-resistant pulmonary tuberculosis patients in Guizhou Province amounted to 10,581.82 RMB ($1453.11), with a median expenditure of 5,277 RMB (IQR: 2,110–12,352 RMB). Notably, indirect expenses comprised 58.07% of the total expenditure. Taking the time of diagnosis as the cut-off point, the majority of these expenses occurred during the treatment phase, but the before diagnosis stage also imposed a significant economic burden, averaging 3,191.58 RMB ($438.27). Among the 2,283 patients, 50.37% (1,150 patients) experienced catastrophic events due to their medical expenses. Key risk factors for these catastrophic events included poverty, employment status, before diagnosis visits, hospitalization, mobility issues, and delayed diagnosis.

**Conclusion:**

The economic burden imposed on households by tuberculosis patients in the province remains considerable, with the indirect burden accounting for the lion’s share. The likelihood of catastrophic expenditures persists, significantly influenced by factors such as poverty, hospitalization, delayed diagnoses, and before diagnosis visits. Recommendations include reinforcing targeted public health education, enhancing the diagnostic and therapeutic capabilities of medical institutions, regulating their practices, curbing unnecessary hospitalizations, and instituting a long-term framework aimed at alleviating the indirect economic burden. By doing so, we can collaboratively diminish the economic strain on patients and mitigate the risk of catastrophic expenditures, ultimately striving for the achievement of zero catastrophic expenditures among households.

## 1 Introduction

Tuberculosis (TB) is a persistent infectious disease stemming from the infection caused by *Mycobacterium tuberculosis*, primarily transmitted via the respiratory tract (1, 2). Due to its vast infection base, heightened drug resistance, intensified population mobility, and prolonged treatment durations, tuberculosis (TB) continues to hold its position as one of the three pivotal diseases initially identified and prioritized for control by the World Health Organization (WHO). Furthermore, it stands as one of the leading ten causes of mortality globally. Not merely that, tuberculosis (TB) bears a profound connection to poverty, potentially inflicting an excruciating economic strain upon patients and their households, while simultaneously precipitating the depletion of vital income streams, thereby plunging families into financial distress (3–5). Hence, TB seriously affects the quality of life of patients and their families and it is also an important public health issue.

In 2014, WHO introduced the “End TB Strategy,” tailored to the prevailing TB burden. This strategy not only articulates two fundamental epidemiological targets—reducing incidence rates and mortality—but also underscores, for the first time, the aspiration to eliminate catastrophic household expenditures, envisioning a TB-free world where TB no longer inflicts death, disease, or suffering upon humanity. This underscores the paramount importance of promptly mitigating the substantial economic burden of TB toward the global eradication of the disease (6, 7).

China ranks as the third-largest country globally burdened with tuberculosis, constituting approximately 7.1% of the total global cases (8). Although the country and government offer free diagnostic tests, anti-tuberculosis medications, and fundamental follow-up services for tuberculosis patients, there remains a significant challenge. At the same time, policies such as covering remaining medical expenses for special poverty - stricken groups like farmers and providing the income losses due to illness have been implemented. But the primary demographic impacted by tuberculosis comprises predominantly farmers residing in economically disadvantaged western regions. Consequently, the additional expenses that the most patient’s family must undertake continue to pose a formidable economic strain on them (9, 10). Based on the findings of the national baseline survey, the age bracket of 15 to 59 years, predominantly male, consistently constitutes the primary demographic group affected by tuberculosis in China. This situation can potentially result in the depletion of productive labor force within affected households, thereby precipitating illness-induced poverty (11).

Guizhou Province, situated in western China, bears a heavy burden of tuberculosis, annually witnessing roughly 40,000 cases being reported (12, 13). The epidemic has consistently ranked third in the country for an extended period. Guizhou Province’s economic development lags behind, and the distribution of tuberculosis cases is notably uneven, predominantly concentrated in economically underdeveloped, impoverished regions and low-income groups, primarily comprising farmers. Among them, roughly one-third of the patients belong to impoverished households, which indicates that the situation of tuberculosis patients in Guizhou Province falling into poverty due to income losses caused by illness and treatment costs incurred because of the disease is relatively common. The pertinent research reports (14) indicate an inadequacy in the number of counties in Guizhou Province implementing medical compensation for tuberculosis, along with the formulated reimbursement standards. Consequently, the out-of-pocket expenses incurred by tuberculosis patients in the province continue to pose an unmanageable economic burden on patients. It is crucial to comprehend the financial weight borne by tuberculosis patients in the province and devise targeted policy proposals aimed at alleviating the burden of tuberculosis. This endeavor holds immense significance not only for the province itself, but also for the nation and indeed, the global community, in our collective quest to halt the tide of tuberculosis epidemic.

Therefore, for the first time in Guizhou Province, this study conducted a comprehensive cross-sectional survey across 89 counties and districts within 9 cities and prefectures, comprehensively measuring the economic burden associated with the diagnosis and treatment of non-drug-resistant pulmonary tuberculosis patients. Concurrently, it rigorously evaluated the catastrophic expenditure scenario outlined by the WHO, delved into the underlying factors influencing this phenomenon, and sought to uncover the potential reasons behind it.

## 2 Methods

### 2.1 Study design and sample

This comprehensive cross-sectional survey was conducted in 89 counties and districts across 9 cities and prefectures within Guizhou Province. The research focused on non-drug-resistant pulmonary tuberculosis patients registered in the tuberculosis registration management system in 2020. These patients had undergone successful treatment, provided informed consent during the survey, and were excluded from the study if they exhibited drug resistance, failed to receive standardized treatment, discontinued treatment, or had communication barriers such as deafness or muteness. Assuming a catastrophic expenditure rate of 70% for tuberculosis patients (15), with a permissible margin of error of 2%, *α* = 0.05, and a non-response rate of 10%, it was necessary to include at least 2,219 cases of pulmonary tuberculosis in the study. To achieve this, we recruited patients from 89 counties and districts across the province, employing a Probability Proportional to Size (PPS) sampling method based on the proportion of registered cases in each county and district in 2020 compared to the total cases in the province. From January to March 2021, a total of 2,521 subjects were surveyed, resulting in the successful recruitment of 2,283 valid participants, achieving a successful response rate of 90.56%.

### 2.2 Study methods

After securing the verbal agreement of the participants, the uniformly trained investigators (the staff engaged in tuberculosis prevention and control at the county - level Centers for Disease Control and Prevention) initially peruse the medical records within the “tuberculosis information management system” to gather pertinent details pertaining to the diagnosis and treatment of the interviewees. Then, the investigator proceeded to enter the residence and conducted face-to-face interviews with the patients, utilizing a structured questionnaire devised in accordance with the survey content endorsed following the WHO‘Patient Cost Survey Handbook’recommendations (16). The objective of these interviews was to gather comprehensive information pertaining to the patients’ medical treatment history and the economic burden they faced both prior to and during the course of the standardized anti-tuberculosis treatment. The designated hospital’s HIS system was utilized by the investigator to inquire about the medical expenses incurred by patients specifically for tuberculosis diagnosis and treatment within the hospital’s premises.

### 2.3 Variable and definitions

#### 2.3.1 Non-drug-resistant pulmonary tuberculosis

The scenario refers to the instance where the *Mycobacterium tuberculosis* infecting the patient has been determined to be non-resistant to the anti-tuberculosis drugs that were tested *in vitro* (17).

#### 2.3.2 The total economic burden of families

Also referred to as the total out-of-pocket expense (OOP), it encompasses the expenses and work-related losses incurred by the patient’s family, spanning from the onset of suspected tuberculosis symptoms to the successful completion of standardized anti-tuberculosis treatment. This encompasses both direct and indirect economic burdens (18). Among them, the direct economic burden pertains to the cumulative expenditure families of tuberculosis patients endure, comprising medical self-payments, accommodations, transportation, and other associated financial pressures incurred as a result of tuberculosis diagnosis and treatment. This burden is further bifurcated into direct medical economic burden, which encapsulates the self-payment medical expenses undertaken by families during the patient’s diagnosis and treatment phase, and direct non-medical economic burden, encompassing transportation, accommodation, nutrition, and other miscellaneous expenses incurred by families during the patient’s diagnostic and therapeutic journey. On the other hand, indirect economic burden alludes to the productivity losses suffered by both patients and their accompanying family members as a consequence of illness-related work absences.

#### 2.3.3 Catastrophic expenditures

Defined as a catastrophic cost based on WHO recommendations, it refers to the situation where the total out-of-pocket economic burden incurred by a patient’s illness exceeds 20% of the household’s annual income (expenditure-to-income ratio) (16). The incidence proportion of catastrophic expenditures refers to the percentage of patient households among the total number that have incurred catastrophic expenditures.

#### 2.3.4 Diagnosis delay

The interval between a patient’s first medical visit for TB symptoms and the confirmed diagnosis exceeding 14 days (19).

#### 2.3.5 Initial treatment of pulmonary tuberculosis

Refers to patients who have not utilized anti-tuberculosis medications, or those who have taken such medications but have undergone treatment for less than a month (20).

#### 2.3.6 Re-treatment of pulmonary tuberculosis

Refers to patients who have experienced initial treatment failure, undergone either regular standard or short-course chemotherapy, and subsequently experienced relapse. Despite undergoing pulmonary resection surgery, new lesions have emerged or worsened, resulting in recurrence (20).

### 2.4 Statistical analysis

Utilized EpiData3.1 software to enter questionnaire details into the database efficiently. Described and analyzed measurement and counting data distinctively, then visually represented them utilizing appropriate charts. Among them, the varying economic burdens were uniformly expressed in RMB. Quantitative data were presented utilizing mean and median (inter-quartile range, IQR), with the Mann–Whitney U test employed for comparing two groups, and the Kruskal-Wallis rank sum test for comparing two or more groups. Counting data were described in terms of proportion, among others, and inter-group comparisons were carried out using the chi-squared (*χ*^2^) test. Fisher’s exact probability method was applied in scenarios where the anticipated frequency of over 20% of the cells fell below 5, or when the projected frequency of a single cell was less than 1. Multivariate analysis was carried out utilizing binary logistic regression. The aforementioned statistical assessments were comprehensively executed within the SPSS 22.0 software platform, employing two-sided tests and maintaining a significance level of 0.05.

### 2.5 Quality control

Pre-investigation stage: This study formulated an initial questionnaire based on the survey manual recommended by the World Health Organization. Additionally, through multiple discussions with numerous experts from the Department of Epidemiology at the School of Public Health, Fudan University, Guizhou Medical University, and the Tuberculosis Prevention and Control Institute of the Guizhou Provincial Center for Disease Control and Prevention, the survey content was determined in accordance with the research objectives of this study and the actual situation of the province, thus ensuring the reliability and credibility of the questionnaire. After two rounds of pre - surveys, the final survey content was confirmed to ensure the feasibility of the survey. Tuberculosis experts from the Guizhou Provincial Center for Disease Control and Prevention provided professional and systematic training to the investigators, ensuring that they had a clear understanding of the survey content.

Investigation stage: When completing the survey, investigators should promptly check for omissions and fill in the gaps to improve the authenticity and effectiveness of the survey. During this period, provincial - level professionals select one county - level district in each city and prefecture for supervision. Subsequently, city - level professionals supervise the survey work within their respective jurisdictions. Any problems identified should be rectified in a timely manner.

Data organization and analysis stage: The double - entry method by two individuals was adopted to input the collected information into the EpiData database. Meanwhile, the collected information was proofread and subjected to logical analysis to ensure the accuracy of the input data. Whenever problems were detected, the person in charge of the county - level district and the investigators were contacted promptly to correct errors and make supplements. Finally, invalid questionnaires with numerous missing items and obvious logical errors were excluded.

## 3 Results

### 3.1 General characteristics of the survey subjects

Among the total of 2,283 patients, the predominant group comprised males, accounting for 61.19% of the total. The most common age group is people aged 21 to 59, accounting for 55.06% of the surveyed population. Middle school education and below constitute a notably high percentage (75.08%). In addition, their distribution varied significantly across various characteristics such as nationality, residence, employment status, and others, with a notable concentration among the Han ethnic group (55.63%), local residents (94.66%), and those who were employed (48.49%), among others.(Table 1).

**Table 1:** General characteristics of the survey subjects.

Indicator	Category	*N* (%)
Sex	Male	1,397 (61.19)
	Female	886 (38.81)
Age (Year)	≤20	413 (18.09)
	21–59	1,257 (55.06)
	≥60	613 (26.85)
Nation	The Han ethnic group	1,270 (55.63)
	Minority nationality	1,013 (44.37)
Education	Uneducated	370 (16.21)
	Elementary school	682 (29.87)
	Middle school	662 (29.00)
	High school	334 (14.63)
	Junior college or above	235 (10.29)
Residence	Local residents	2,161 (94.66)
	Migrants	122 (5.34)
employment before TB diagnosis	Student	417 (18.27)
	Employed	1,107 (48.49)
	Other	759 (33.25)
Medical insurance	Urban employee basic medical insurance (UEBMI)	119 (5.21)
	Urban–rural resident medical insurance (URRMI)	1,617 (70.83)
	Urban resident basic medical insurance (URBMI)	64 (2.80)
	New rural cooperative medical scheme (NRCMS)	474 (20.76)
	Other insurance	9 (0.39)
Major labor force	Yes	937 (41.04)
	No	1,346 (58.96)
Phlegm test results	Negative	1,123 (49.19)
	Positive	1,136 (49.76)
	Not detected	24 (1.05)
Patient type	Initial treatment	2,237 (97.99)
	Re-treatment	46 (2.01)
Merge additional tuberculosis	Yes	33 (1.45)
	No	2,250 (98.55)
Co-existing with other chronic diseases	No	1818 (79.63)
	Yes	465 (20.37)
Hospital visits before diagnosis	No	591 (25.89)
	Yes	1,692 (74.11)
Delayed diagnosis	Yes	701 (30.71)
	No	1,582 (68.29)
Hospitalization history	No	847 (37.10)
	Yes	1,436 (62.90)
Annual household income*	Q1	483 (21.16)
	Q2	472 (20.67)
	Q3	440 (19.27)
	Q4	555 (24.31)
	Q5	333 (14.59)

*Q1 ~ 5: the range of annual household income, spanning from the lowest quintile (Q1) to the highest (Q5).

### 3.2 Economic burden and composition of pulmonary tuberculosis patients

The average total out-of-pocket expenses (OOP) for 2,283 pulmonary tuberculosis patients amounted to 10,581.82 RMB ($1453.11), with a median value (IQR) of 5,277 RMB, ranging from 2,110 to 12,352 RMB. Among the various categories, 32.20% comprised the direct medical burden, with an average of 3,407.10 RMB ($467.87) and a median (IQR) of 1,995 (916–4,058) RMB. 9.73% constituted direct non-medical burden, with an average cost of 1,029.49 RMB ($141.37), while the Median (IQR) stood at 690 RMB (252–1,400 RMB). 58.07% comprised indirect burden, with a mean value of 6,145.22 RMB ($19.94) and a median (IQR) of 555 RMB (0 to 6,000 RMB). The noteworthy statistical significance lies in the disparities among these three economic burdens (*χ*^2^ = 639.368, *p* < 0.001).

**Figure 1:**
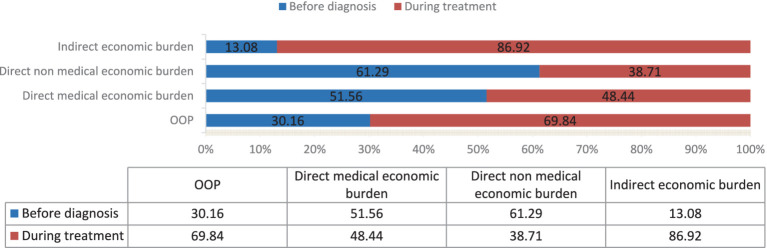
The distribution of patient economic burden at different treatment stages.

Using the time of diagnosis as the dividing line, the OOP for patients prior to diagnosis averaged 3,191.58 RMB ($438.27), with a median (IQR) of 1,235(0–3,677) RMB. The average of the OOP for patients undergoing anti-tuberculosis treatment period amounted to 7,390.23 RMB ($1014.83), while the median (IQR) stood at 2,580 RMB (976–8,051 RMB). The statistical significance of the disparity between the two sides is undeniable (*Z* = -19.981, *p* < 0.001). Among them, during the before diagnosis stage, the majority of the incurred expenses are primarily direct medical expenses (*χ*^2^ = 652.021, *p* < 0.001), while indirect expenses became the majority during the treatment stage (*χ*^2^ = 498.881, *p* < 0.001) (Table 2 and Figure 1).

**Table 2:** Various expenses and distribution.

Indicator	The entire stage			Before the diagnosis stage			During the treatment stage		
	Mean	Median (IQR)	%	Mean	Median (IQR)	%	Mean	Median (IQR)	%
1. OOP	10581.82	5,277 (2110–12,352)	100.00	3191.58	1,235 (0–3,677)	100.00	7390.23	2,580 (976–8,051)	100.00
2. Direct economic burden	4436.59	2,924 (1366–5,440)	41.93	2387.69	1,050 (0–2,960)	74.81	2048.90	1,149 (613–2,330)	27.72
2.1. Direct medical economic burden	3407.10	1995 (916–4,058)	32.20	1756.69	697 (0–2,960)	55.04	1650.41	816 (330–1826)	22.33
2.2. Direct non medical economic burden	1029.49	690 (252–1,400)	9.73	630.99	199 (0–900)	19.77	398.50	240 (100–500)	5.39
2.2.1. Transportation expenditures	331.83	240 (110–450)		98.19	36 (0–100)		233.65	160(60–300)	
2.2.2. Food and accommodation expenditures	533.78	280 (0–750)		414.06	0 (0–0)		119.72	0 (0–180)	
2.2.3. Other	163.90	0 (0–200)		118.74	0 (0–130)		45.16	0 (0–0)	
3. Indirect economic burden	6145.22	555 (0–6,000)	58.07	803.89	0 (0–340)	25.19	5341.33	0 (0–5,359)	72.28
3.1. Patient	4147.10	0 (0–4,000)		218.54	0 (0–33)		3928.56	0 (0–3,200)	
3.2. Family members	1998.12	0 (0–980)		585.34	0 (0–100)		1412.77	0 (0–300)	

### 3.3 Economic burden allocation among patients with diverse characteristics

The composition of direct medical burden, direct non medical burden and OOP varied significantly across patients with diverse characteristics. The direct medical burden, direct non medical burden and OOP of mobile patients were higher than those of local patients (*Z* = −6.083, −3.408, −4.125, all *p* < 0.05). The various burden of employed patients were higher than those of other patients (*χ*^2^ = 15.604, 10.602, 187.926, 153.124, all *p* < 0.05). The various burden of patients who visits before diagnosis were higher than those of patients who did not (*Z* = −17.485, −19.031, −7.301, −14.041, all *p* < 0.05). The various burden of patients with delayed diagnosis were higher than those of non-delayed diagnosis patients (*Z* = −15.301, −14.360, −6.904, −13.148, all *p* < 0.05). The various burden of hospitalized patients were higher than those of non hospitalized patients (*Z* = −24.662, −23.185, −6.794, −17.559, all *p* < 0.05). The differences in various burden among different income groups were also statistically significant (*χ*^2^ = 31.627, 4.778, 15.998, 37.924, all *p* < 0.05) (Table 3).

**Table 3:** Economic burden allocation among patients with diverse characteristics.

Indicator					Direct economic burden			Indirect economic burden			OOP	
		Direct medical economic burden			Direct non medical economic burden							
		Mean	Median (IQR)	%	Mean	Median (IQR)	%	Mean	Median (IQR)	%	Mean	Median (IQR)
Sex	Male	3436.99	1995 (913–4,059)	30.91	1043.82	705 (270–1,400)	9.39	6637.29	800 (0–6,590)	59.70	11118.10	5,539 (2251–13,125)
	Female	3359.98	1991 (919.65–4,035)	34.51	1006.90	651 (240–1,400)	10.34	5369.36	300 (0–5,312)	55.15	9736.24	4,939 (1872–11,032)
Age (Year)	≤20	2547.61	1,673 (854–3,046)	33.03	996.92	670 (240–1,314)	12.93	4168.55	0 (0–2008)	54.05	7713.09	3,460 (1497–6,908)
	21–59	3564.22	2028 (877–4,380)	27.70	950.59	606 (228–1,325)	7.39	8352.10	1,400 (0–10,420)	64.91	12866.91	6,570 (2339–16,239)
	≥60	3663.98	2,194 (1017–4,200)	46.80	1213.22	850 (350–1,570)	15.50	2951.63	200 (0–3,225)	37.70	7828.82	4,910 (2288–10,033)
Nation	The Han nationality	3731.30	2,253 (1047–4,661)	32.34	1093.75	740 (300–1,502)	9.48	6711.18	700 (0–6,465)	58.17	11536.22	5,924 (2519–13,467)
	Minority nationality	3000.65	1719 (804–3,416)	31.97	948.94	650 (205–1,298)	10.11	5435.69	400 (0–5,625)	57.92	9385.28	4,666 (1750–11,130)
Education	Uneducated	2847.79	1,680 (904–3,201)	36.83	1129.88	800 (338–1,425)	14.61	3754.60	560 (0–4,155)	48.56	7732.26	4,494 (1859–9,280)
	Primary school	3995.50	2,265 (987–4,875)	30.77	1174.49	760 (300–1,631)	9.05	7813.19	1,170 (0–7,600)	60.18	12983.18	6,594 (2509–14,851)
	Middle school	3243.59	1963 (906–3,735)	26.94	1008.86	680 (240–1,403)	8.38	7786.38	1,047 (0–9,168)	64.68	12038.83	5,993 (2579–14,554)
	High school	2858.58	1750 (849–3,720)	37.36	832.25	540 (192–1,100)	10.88	3960.20	0 (0–2,900)	51.76	7651.03	3,918 (1598–8,888)
	Junior college or above	3820.32	2073 (906–4,666)	46.82	789.11	440 (120–1,010)	9.67	3550.88	0 (0–2,800)	43.51	8160.31	4,145 (1462–9,183)
Residence	Local patient	3243.30	1920 (900–3,832)	31.36	1038.58	704 (270–1,410)	10.04	6058.54	560 (0–5,957)	58.59	10340.42	5,100 (2047–12,058)
	Mobile patient	6308.46	4,223 (1919–8,426)	42.46	868.59	405 (120–1,508)	5.85	7680.66	412 (0–11,286)	51.69	14857.71	8,656 (3886–20,637)
Employment before TB diagnosis	Student	2488.51	1,647 (849–2,993)	37.38	936.62	610 (196–1,213)	14.07	3232.53	0 (0–1,420)	48.55	6657.66	3,007 (1388–6,128)
	Employed	3656.21	2,122 (962–4,356)	26.80	994.62	652 (270–1,350)	7.29	8989.30	2,700 (0–11,880)	65.90	13640.14	7,156 (3003–17,902)
	Other	3548.45	2,100 (936–4,375)	42.87	1131.37	760 (270–1,560)	13.67	3597.39	100 (0–3,174)	43.46	8277.22	4,526 (1860–10,626)
Medical insurance	UEBMI	4885.92	2,646 (1203–6,458)	50.11	957.81	500 (180–1,050)	9.82	3906.72	0 (0–4,000)	40.07	9750.45	6,347 (2202–12,012)
	URRMI	3447.37	2058 (982–4,125)	31.49	1088.54	740 (300–1,525)	9.94	6411.13	692 (0–6,205)	58.56	10947.04	5,539 (2200–12,861)
	URBMI	2756.96	1,430 (460–3,446)	33.37	724.31	210 (26–959)	8.77	4781.19	190 (0–4,893)	57.87	8262.47	3,942 (1338–7,795)
	NRCMS	2970.97	1708 (755–3,283)	29.94	891.78	600 (206–1,232)	8.99	6061.84	700 (0–6,082)	61.08	9924.59	4,633 (1744–11,816)
	other insurance	4211.51	2,231 (1054–4,991)	59.63	791.22	330 (12–1,510)	11.20	2060.00	0 (0–1995)	29.17	7062.73	2,231 (1227–14,465)
Major labor force	Yes	3592.26	2096 (921–4,335)	27.54	981.71	606 (243–1,340)	7.53	8468.31	1800 (0–10,795)	64.93	13042.28	6,810 (2561–16,550)
	No	3278.20	1924 (909–3,860)	36.96	1062.76	740 (260–1,433)	11.98	4528.04	176 (0–3,663)	51.05	8869.00	4,516 (1865–9,907)
Phlegm test results	Negative	3298.34	1850 (847–3,889)	30.19	1014.66	650 (220–1,400)	9.29	6611.46	392 (0–5,940)	60.52	10924.46	4,925 (1916–11,760)
	Positive	3477.15	2,117 (987–4,085)	34.05	1041.80	715 (300–1,400)	10.2	5691.60	708 (0–6,208)	55.74	10210.54	5,784 (2219–12,863)
	Not detected	5180.68	4,361 (2666–7,164)	42.74	1141.00	645 (278–1,405)	9.41	5800.92	0 (0–9,695)	47.85	12122.60	7,041 (4376–16,791)
Patient type	Initial treatment	3413.37	1995 (917–4,056)	32.45	1022.61	680 (250–1,385)	9.72	6083.53	500 (0–6,000)	57.83	10519.51	5,200 (2102–12,276)
	Re-treatment	3102.17	1992 (767–4,499)	22.79	1364.39	1,075 (405–1895)	10.02	9145.46	2,653 (0–13,162)	67.19	13612.02	9,194 (3248–17,563)
Merge additional tuberculosis	Yes	7109.28	2,866 (816–5,708)	31.45	1382.91	840 (295–1708)	6.12	14114.15	3,375 (0–15,490)	62.43	22606.34	7,820 (2750–27,543)
	No	3352.80	1989 (918–4,051)	32.22	1024.31	686 (250–1,400)	9.84	6028.35	509 (0–6,000)	57.93	10405.46	5,217 (2099–12,274)
Co-existing with other chronic diseases	No	3181.34	1813 (857–3,783)	30.60	979.35	650 (240–1,340)	9.42	6236.88	449 (0–6,000)	59.98	10397.58	5,013 (1938–11,839)
	Yes	4289.19	2,770 (1379–5,233)	37.95	1225.53	844 (341–1,631)	10.84	5786.87	800 (0–11,839)	51.20	11302.14	6,372 (2807–13,702)
Visits before diagnosis	No	1649.60	919 (330–2068)	25.99	431.77	255 (108–600)	6.80	4265.64	0 (0–4,200)	67.21	6347.00	2,238 (996–6,908)
	Yes	4020.82	2,376 (1317–4,876)	33.38	1238.27	900 (400–1,650)	10.27	6801.75	1,000 (0–6,749)	56.39	12061.00	6,390 (2978–14,065)
Delayed diagnosis	Yes	5209.86	3,351 (1682–6,597)	33.62	1534.87	1,140 (540–2080)	9.90	8752.51	2057 (0–9,449)	56.48	15497.24	9,050 (4110–18,462)
	No	2608.28	1,557 (749–3,040)	31.04	805.56	527 (200–1,100)	9.59	4989.91	185 (0–5,040)	59.38	8403.74	4,031 (1637–9,484)
Hospitalization history	No	1507.76	876 (360–1732)	23.75	456.98	300 (120–596)	7.20	4384.70	0 (0–5,000)	69.06	6349.44	2,300 (938–7,090)
	Yes	4527.39	2,822 (1597–5,405)	34.62	1367.18	1,040 (530–1815)	10.45	7183.64	1,164 (0–6,920)	54.93	13078.21	6,966 (3518–14,474)
Annual household income	Q1	3065.61	1942 (900–3,799)	34.99	1085.43	740 (300–1,530)	12.39	4611.52	400 (0–4,880)	52.63	8762.56	4,699 (1995–10,129)
	Q2	2948.17	1750 (843–3,559)	32.69	959.41	645 (250–1,338)	10.64	5112.00	400 (0–5,243)	56.68	9019.59	4,711 (1752–10,580)
	Q3	3068.68	1718 (753–3,329)	35.97	947.32	640 (240–1,305)	11.10	4514.95	295 (0–5,201)	52.92	8530.95	4,554 (1765–10,711)
	Q4	4067.76	2,200 (1080–4,948)	34.52	1092.01	704 (250–1,530)	9.27	6624.48	800 (0–8,883)	56.21	11784.25	6,284 (2377–15,038)
	Q5	3898.98	2,402 (1124–4,771)	24.16	1052.06	720 (235–1,300)	6.52	11189.64	0 (1204–9,863)	69.33	16140.67	6,502 (2916–17,767)

### 3.4 Proportion of out-of-pocket (OOP) expenses relative to household income

**Figure 2:**
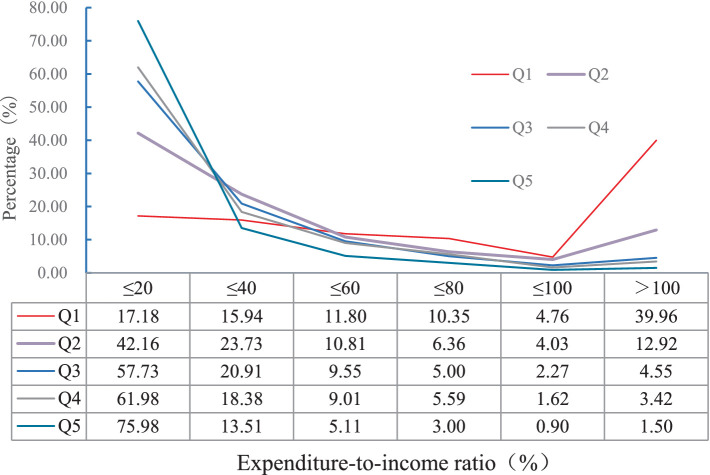
Distribution of the proportion of total out of pocket expenses to annual household income for patients with different household incomes.

Categorized the family units into five distinct strata (Q1-Q5), each representing a quintile of annual income, wherein the lowest quintile (Q1) is designated as “economically disadvantaged families” and the highest quintile (Q5) is labeled as “financially prosperous families.” The expenditures incurred by patients belonging to financially prosperous families were markedly greater than those from economically disadvantaged families, yet when it comes to the proportion of OOP relative to annual household income, patients from economically disadvantaged families displayed significantly higher percentages than those from financially prosperous families. Among patients hailing from financially prosperous families, 24.02% exhibited an expenditure-to-income ratio surpassing 20%, whereas a staggering 82.82% of patients from financially prosperous families demonstrated an expenditure-to-income ratio exceeding the same threshold (Figure 2).

### 3.5 Catastrophic expenditures and influencing factors

The proportion of catastrophic expenditures incurred by 2,283 patients stood at 50.37%, specifically 1,150 out of the total 2,283 patients. The statistically significant differences in the proportion of catastrophic expenditures experienced by patients of varying ages, nation, education, residence, employment statuses, medical insurance, whether they constitute the major labor force, patient types, co-existence with other chronic diseases, visit before diagnosis, delayed diagnosis, hospitalization histories, and annual family incomes (before TB diagnosis) are noteworthy (all *p* < 0.05). Furthermore, utilizing whether the patient’s family incurred catastrophic expenses as the dependent variable (coded as 0 for ‘no’ and 1 for ‘yes’), the aforementioned statistically significant variables were incorporated into the multivariate logistic regression analysis. The results showed that economically disadvantaged families (*OR* = 30.111, 95% *CI*: 2.048–45.227), employed (*OR* = 3.821, 95% *CI*: 2.280–6.404), visits before diagnosis (*OR* = 3.153, 95% *CI*: 2.454–4.052), hospitalization (*OR* = 2.724, 95% *CI*: 2.156–3.443), mobility (*OR* = 2.320, 95% *CI*: 1.461–3.686) and delayed diagnosis (*OR* = 2.221, 95% *CI*: 1.774–2.781) were risk factors for catastrophic expenditures in patient families (all *p* < 0.05) (Table 4).

**Table 4:** Catastrophic expenditures among the patient’s family.

Indicator		Number of patients	Number of catastrophic expenditures patients	Catastrophic expenditures rate (%)	*χ*^2^ test		Multivariate logistic regression	
					*χ*2	*P*	OR (95*%*CI)	*P*
Sex	Male	1,397	716	51.25	1.116	0.291	-	
	Female	886	434	48.98			-	
Age (Year)	≤20	413	130	31.48	72.014	<0.001	1	
	21–59	1,257	686	54.57			0.824 (0.511–1.328)	0.427
	≥60	613	334	54.49			0.600 (0.350–1.029)	0.064
Nation	Minority nationality	1,013	461	45.51	17.233	<0.001	1	
	the Han nationality	1,270	689	54.25			1.221 (0.993–1.501)	0.059
Education	Junior college or above	235	77	32.77			1	
	High school	334	112	33.53	95.658	<0.001	1.113 (0.715–1.734)	0.635
	Middle school	662	350	52.87			1.546 (1.033–2.313)	0.034
	Primary school	682	407	59.68			1.591 (1.038–2.440)	0.033
	Uneducated	370	204	55.14			1.183 (0.727–1.927)	0.499
Residence	Local patient	2,161	1,074	49.70	7.329	0.007	1	
	Mobile patient	122	76	62.30			2.320 (1.461–3.686)	<0.001
employment before TB diagnosis	Student	417	112	26.86	120.419	0.007	1	
	Employed	1,107	645	58.27			3.821 (2.280–6.404)	<0.001
	Other	759	393	51.78			1.813 (1.066–3.085)	0.004
Medical insurance	UEBMI	119	46	38.66	11.392	0.022	1	
	URRMI	1,617	844	52.20			1.230 (0.756–2.002)	0.404
	URBMI	64	27	42.19			1.252 (0.578–2.713)	0.569
	NRCMS	474	229	48.31			0.981 (0.579–1.663)	0.943
	other insurance	9	4	44.44			1.414 (0.236–8.461)	0.704
Major labor force	Yes	937	548	58.48	41.837	<0.001	1	
	No	1,346	602	44.73			1.263 (0.987–1.1.615)	0.063
Phlegm test results	Negative	1,123	545	48.53	3.044	0.218	-	
	Positive	1,136	593	52.20			-	
	Not detected	24	12	50.00			-	
Patient type	Initial treatment	2,237	1,118	49.98	6.918	0.009	1	
	Re-treatment	46	32	69.57			1.645 (0.735–3.678)	0.226
Merge additional tuberculosis	Yes	33	21	63.64	2.357	0.125	-	
	No	2,250	1,129	50.18			-	
Co-existing with other chronic diseases	No	1818	868	47.74	23.835	<0.001	1	
	Yes	465	281	60.43			1.171 (0.902–1.519)	0.236
Visits before diagnosis	No	591	222	37.56	52.336	<0.001	1	
	Yes	1,692	928	54.85			1.903 (1.481–2.444)	<0.001
Delayed diagnosis	No	1,582	704	44.50	71.057	<0.001	1	
	Yes	701	446	63.62			2.089 (1.666–2.619)	<0.001
Hospitalization history	No	847	285	33.65	150.663	<0.001	1	
	Yes	1,436	865	60.24			3.237 (2.594–4.038)	<0.001
Annual household income	Q5	333	80	24.02	351.804	<0.001	1	
	Q4	555	211	38.02			2.432 (1.732–3.415)	<0.001
	Q3	440	186	42.27			3.523 (2.462–5.040)	<0.001
	Q2	472	273	57.84			7.639 (5.328–10.953)	<0.001
	Q1	483	400	82.82			32. (21.480–48.923)	<0.001

## 4 Discussion

The average OOP for 2,283 non-drug-resistant pulmonary tuberculosis patients across 89 counties and districts in Guizhou Province amounted to 10,581.82 RMB ($1453.11), with a median of 5,277 RMB, this figure surpasses that of other domestic and international locations (21–24). For example, in a survey conducted in Xinjiang, the total out-of-pocket expenses for 251 pulmonary tuberculosis patients averaged 9,291.7 RMB per person, with a median of 3,949.4RMB. In a survey conducted in Taixing City, Jiangsu Province, the average total out-of-pocket expenses per person for 316 pulmonary tuberculosis patients was 5,639.2RMB. Potentially attributable to disparities in economic levels, diagnosis and treatment methods, medical reimbursement rates, and various other factors among different regions. Simultaneously, the incidence proportion of catastrophic expenditures stood at 50.37%, it is comparable to the 52% obtained from the investigation conducted in 16 designated tuberculosis medical institutions in 12 provinces in the western region of our country (25), markedly deviating from the World Health Organization’s (WHO) “End TB Strategy” objective of achieving zero catastrophic household expenditures. This signifies that the risk of poverty resulting from tuberculosis remains considerable in Guizhou Province. To attain the ambition of eradicating the prevalence of tuberculosis, it is crucial to alleviate the financial burden imposed on tuberculosis patients and mitigate the devastating expenditures endured by their families. This issue necessitates urgent attention and concerted efforts from the government and pertinent departments. Therefore, it is imperative to delve deeper into the prevailing circumstances and potential factors contributing to the economic burden borne by pulmonary tuberculosis patients within the province, with the aim of refining and enhancing the tuberculosis prevention and control strategies and measures implemented therein.

In prior research, the total economic burden on patients has primarily been direct economic burden, notably with the preponderance of direct medical economic comprising the lion’s share (18, 22, 26). Among the total economic burden incurred by 2,283 pulmonary tuberculosis patients, the indirect economic burden constituted a significant 58.07%, while the direct medical economic burden comprised 32.20% of the total economic burden. There may be various reasons contributing to this. Firstly, under the keen focus of governments at all levels and pertinent departments, investments in tuberculosis prevention and control have been escalating. This has entailed the allocation of funds specifically for the unified procurement of first-line anti-tuberculosis medications across the province, thereby ensuring the entire course of free treatment for non-drug-resistant patients with these medications. The related studies (18, 27) have demonstrated that decreasing drug expenses is crucial for alleviating the direct economic burden. Additionally, the funding guarantee not only ensured free tuberculosis diagnosis and testing for patients, but also covered a portion of the essential follow-up examinations throughout their entire treatment journey. The second factor is the expansion of reimbursement scope and the increase of proportion to reduce the economic burden related to diagnosis and treatment of tuberculosis in the province (28). Thirdly, the province implemented proactive tuberculosis detection measures, actively screening close contacts of confirmed patients, students, and other high-risk groups. This enhancement led to an improved level of patient identification, subsequently reducing instances of patients making repeated visits and experiencing delays in seeking medical attention. Fourthly, at present, there exists no policy for compensating the indirect economic losses incurred by the treatment of pulmonary tuberculosis patients. While ensuring compensation for the medical economic burden faced by patients, addressing the indirect economic burden holds the key to further alleviating the total disease burden on them.

The overall economic burden was predominantly (69.84%) incurred during the treatment phase of patients’ care, aligning with findings from other research studies (29). However, the before diagnosis phase imposes a significant economic burden (30.16%), where the direct economic burden surpasses that of the indirect one. Possible correlation with low public awareness regarding tuberculosis (30), resulting in a frequent occurrence of repeated visits and delayed diagnosis prior to confirmation. Additionally, it was correlated with the decreased reimbursement ratio that medical institutions offered for patient diagnosis and treatment expenses incurred prior to the actual diagnosis. Further enhancing health education for the public and improving the level of diagnosis and treatment in medical institutions is imperative.

This study further established that economically disadvantaged families, employed, visits before diagnosis, hospitalization, mobility and delayed diagnosis are all risk factors contributing to catastrophic expenditures among patient families. In the composition of economic burdens, despite the fact that the diverse expenses incurred by economically disadvantaged are relatively lower than those of financially prosperous ones, their vulnerability to catastrophic expenditures remains significantly higher. It is suggested that the expenditures caused by tuberculosis is still an important expenditure for the backward social and economic level. Although the expenditures are not higher than those of other sides, the risk of poverty following the onset of the disease is widespread due to the society’s inadequate payment capabilities (31–33). The main reason for the higher risk of catastrophic expenditures for employed patients was that they suffered more losses due to illness, which was consistent with the results of this study where the indirect economic burdens of in-service patients were significantly higher than those of other patients (34). Related researchers have held the belief (34–38) that owing to limitations in the diagnostic and treatment capabilities of doctors and medical institutions, patients faced delays in diagnosis and repeatedly incurred various expenditures prior to the commencement of anti-tuberculosis treatment. Especially, it has been discovered that approximately half of patients undergo hospitalization prior to anti-tuberculosis treatment, and their treatment costs were higher. Certain scholars contend that significant hospitalization rates, coupled with elevated hospitalization costs, constitute pivotal factors influencing the economic burden imposed upon patients (5, 31, 39). In this survey, an overwhelming 71.62% of patients were admitted to hospitals due to their illnesses throughout the entire period, posing a significant risk factor for incurring catastrophic medical expenses. The nationally established guidelines for tuberculosis prevention and control explicitly state that the primary mode of treatment for tuberculosis patients should revolve around outpatient services, while reserving hospitalization solely for those with severe illness (20). On the one hand, it underscores the paramount significance of enhancing the precision diagnosis capabilities of medical institutions. On the other, it underscores the imperative need for regulating diagnostic and treatment practices to curb unwarranted hospitalizations.

This study was grounded on the WHO primary emphasis on the aspirational goal of achieving zero catastrophic household expenditures. For the first time ever, an extensive survey was undertaken among tuberculosis patients residing in 89 counties and districts across the entire province. The objective was to gain a comprehensive understanding of the economic burden and catastrophic expenditure patterns of these patients within the province, thereby furnishing a scientific foundation for refining and enhancing prevention and control strategies within the region. However, its limitations reside in: firstly, the participants of this survey were solely successfully treated pulmonary tuberculosis patients, and the extended treatment duration (6 months) potentially introduces recall bias. Secondly, there may exist patients facing economic hardships who are unable to persist in completing the entire treatment process, thereby leading to potential selection bias. Thirdly, owing to the extended treatment duration spanning 18 to 24 months and the elevated financial costs associated with drug-resistant pulmonary tuberculosis patients, the quantity of successfully treated individuals within the province was not only limited but also dispersed. Consequently, these patients were excluded from the current survey, potentially resulting in an underestimation of the economic burden and the proportion of catastrophic expenditures ultimately incurred by the patients.

In conclusion, the risk of catastrophic expenditures among families of tuberculosis patients in Guizhou Province remains notably prevalent. Key risk factors contributing to these devastating household expenses include poverty, hospitalization, delayed diagnosis, and before diagnosis visits for TB patients. In conclusion, the risk of catastrophic expenditures among families of tuberculosis patients in Guizhou Province remains notably prevalent. Key risk factors contributing to these devastating household expenses include poverty, hospitalization, delayed diagnosis, and before diagnosis visits for TB patients. The second imperative is to bolster the training and oversight of medical institutions, thereby enhancing the standard of diagnosis and treatment, standardizing medical practices, guaranteeing prompt and standardized patient care, and mitigating the incidence of unwarranted hospital admissions. The third is to actively seek funding from diverse directions and departments, striving for elevated levels of medical compensation for patients, and ensuring comprehensive social assistance is provided to them. Develop a sustainable mechanism aimed at alleviating indirect economic pressures, collaboratively mitigating the financial stress on patients, and diminishing the likelihood of catastrophic expenditures, thereby striving toward the attainment of zero catastrophic household expenditures.

## Data Availability

The original contributions presented in the study are included in the article/supplementary material, further inquiries can be directed to the corresponding authors.

## References

[1] Chisompola NK Streicher EM Dippenaar A Whitfield MG Tembo M Mwanza S. Drug resistant tuberculosis cases from the Copperbelt province and northern regions of Zambia: genetic diversity, demographic and clinical characteristics. Tuberculosis. (2021) 130:102122. doi: 10.1016/j.tube.2021.102122, PMID: 34517268

[2] World Health Organization. Global tuberculosis report 2020. Geneva: World Health Organization (2020).

[3] Zeng Y Yang X Zhou H Pu L. Disease burden of tuberculosis in the chines population: a systematic review. Chin J Evid Based Med. (2018) 18:570–9. doi: 10.7507/1672-2531.201801013

[4] Xu B. Regulating tuberculosis medical care and achieving no affected families facing catastrophic costs due to tuberculosis. Chin J Antitubere. (2019) 41:485–7. doi: 10.3969/j.issn.1000-6621.2019.05.004

[5] Wang Q Wang L Li R Ruan Y Chen M Sun Q. Analysis of the medical expenses and economic burden of pulmonary tuberculosis patients in three cities. Chin J Antitubere. (2013) 35:240–5. doi: 10.19982/j.issn.1000-6621.2013.04.004

[6] World Health Organization. Global strategy and goals for TB prevention, treatment and control after 2015. Geneva: World Health Organization (2014).

[7] World Health Organization. The end TB strategy. Geneva: World Health Organization (2015).

[8] World Health Organization. Global tuberculosis report 2023. Geneva: World Health Organization (2023).

[9] Qiu S Pan H Zhang S Peng X Zheng X Xu G. Is tuberculosis treatment really free in China? A study comparing two areas with different management models. PLoS One. (2015) 10:e0126770. doi: 10.1371/journal.pone.0126770, PMID: 25993411 PMC4439067

[10] Jiang S Wang L. Target - "zero" patients with catastrophic household expenditure due to tuberculosis. Chin J Antitubere. (2016) 6:425–7. doi: 10.3969/j.issn.1000-6621.2016.06.001

[11] Wang L Cheng S Chen M Zhao Y Zhang H Jiang S. The fifth national tuberculosis epidemiological survey in 2010. Chin J Antitubere. (2012) 34:485–508. doi: 10.19982/j.issn.1000-6621.2012.08.001

[12] Zhou J. Analysis of the epidemic characteristics and diagnosis and treatment of drug resistant pulmonary tuberculosis in Guizhou Province from 2013 to 2018. Guiyang, Guizhou Province, China:Guizhou Medical University (2021).

[13] Ma X Zhou J Tian J Zhou J Guo X Chen H. Spatial and temporal distribution characteristics of pulmonary tuberculosis in Guizhou Province from 2015 to 2020. Modern Prevent Med. (2021) 48:3415–20. doi: 10.20043/j.cnki.mpm.2021.18.033

[14] Chen Z Yang J Chen H. Investigation and analysis of medical compensation for diagnosis and treatment of pulmonary tuberculosis in Guizhou province. Guizhou Med J. (2012) 36:56–7. doi: 10.3969/j.ISSN.1000-744X.2012.01.025

[15] Xiang L Pan Y Hou S Zhang H Sato KD Li Q. The impact of the new cooperative medical scheme on financial burden of tuberculosis patients: evidence from six counties in China. Infect Dis Poverty. (2016) 5:8. doi: 10.1186/s40249-015-0094-5 26818723 PMC4730613

[16] World Health Organization. Tuberculosis patient CostSurveys: A handbook. Geneva: World Health Organization (2017).

[17] The National Health and Family Planning Commission of the People's Republic of China, *WS 196–2017 classification of tuberculosis*. (2017). https://www.ndcpa.gov.cn/jbkzzx/crb/common/content/content_1656311639939289088.html

[18] Liu Y Xu CH Wang ZY Wang XM Wang YH Zhang H. A cross-sectional study on economic burden of pulmonary tuberculosis cases from designated tuberculosis hospital. Chin J Epidemiol. (2019) 5:559–64. doi: 10.3760/cma.j.issn.0254-6450.2019.05.013 31177738

[19] Hong CY Wang FL Zhang YT Tao FX Ji LC Lai PX. Time-trend analysis of tuberculosis diagnosis in Shenzhen, China between 2011 and 2020. Front Public Health. (2023) 11:1059433. doi: 10.3389/fpubh.2023.1059433, PMID: 36891348 PMC9986421

[20] Tuberculosis prevention and control Center of China Center for disease control and prevention, technical guidelines for tuberculosis prevention and control in China. Beijing: People's Health Publishing House (2021). https://www.chinacdc.cn/jkyj/crb2/yl/fjh/jswj_fjh/202410/P020241010432930570191.pdf

[21] Kilale AM Pantoja A Jani B Range N Ngowi BJ Makasi C. Economic burden of tuberculosis in Tanzania: a national survey of costs faced by tuberculosis-affected households. BMC Public Health. (2022) 22:600. doi: 10.1186/s12889-022-12987-3 35351063 PMC8961947

[22] Du S., Investigation on the current situation and influencing factors of economic burden of tuberculosis patients in five counties and districts of Xinjiang, Tuberculosis Prevention and Control Center of China Center for Disease Control and Prevention. (2018). https://lxr.gmc.edu.cn/https/webvpn34dba54512b1dbccec764ab274be469e/kcms2/article/abstract?v=TD_mLQSGK6uQbSdmHve4BCSDXEFI8Ry-QyUFONLdJGk8mkvRifAUJtF0DP7U_nAcUG5kybpqiEfQ7RIB4BIQAVY5UxwyS6FxY8KC_j-1vEp1fGvrd9ziDryuTezF2-c9VwtluRhnjhEEU7KIVQazLPyFK_ouXyS-IxdISDGFJ7RNAwxFy1F6bg00nk77Ng2V&uniplatform=NZKPT&language=CHS

[23] Qiu S LLu H Zhang S Jiang W Huang L Wang J. Comparative study on economic burden of tuberculosis patients. J Nanjing Med Univ. (2014) 14:54–8. doi: 10.7655/NYDXBSS20140503

[24] Kaswa M Minga G Nkiere N Mingiedi B Eloko G Nguhiu P. The economic burden of TB-affected households in DR Congo. Int J Tuberc Lung Dis. (2021) 25:923–32. doi: 10.5588/ijtld.21.0182, PMID: 34686235 PMC8544924

[25] Hao D Li T Huang F Xu C. A cross-sectional study on the economic burden of pulmonary tuberculosis patients from western China. Chin J Antitubere. (2023) 45:1021–30. doi: 10.19982/j.issn.1000-6621.20230137

[26] World Health Organization. Global monitoring report on financial protection in health 2021. Geneva: World Health Organization (2021).

[27] Wang W Shen X Wang W Guo M Wu Z Chen J. Outpatient direct medical expenditures and their composition of patients with pulmonary tuberculosis in Shanghai. Med Soc. (2021) 34:25–30. doi: 10.13723/j.yxysh.2021.07.006

[28] Guo X Chen H Li Y Ma X Zhou J Zhou J. Investigation and analysis of the current situation of medical coverage for tuberculosis in Guizhou Province. Modern Prevent Med. (2022) 49:3374–8. doi: 10.20043/j.cnki.MPM.202204441

[29] Huang F Wang L Yang H Bai L. Analysis of cost for TB patients in 10 counties, Hunan. Chin J Antitubere. (2009) 31:449–53. doi: 10.19982/j.issn.1000-6621.2009.08.003

[30] Zhang R He L Li Y Yu X Yu H. Analysis on the public’s awareness about tuberculosis key information in Guizhou Province. Chin J of Health Education. (2019) 35:592–5. doi: 10.16168/j.cnki.issn.1002-9982.2019.07.004

[31] Shi O Hou W Yang R Li W Xu H Lu Z. The analysis of treatment cost and its influencing factors of tuberculosis patients in communities. Wuhan Med Soc. (2012) 25:38–40. doi: 10.3870/YXYSH.2012.07.014

[32] Yang X Zhou W Ji D Lu X Yang C. Analysis of economic burden and influencing factors OD pulmonary tuberculosis patents in Yangcheng in 2015-2016. Chin J Soc Med. (2019) 4:430–3. doi: 10.3969/j.issn.1673-5625.2019.04.026

[33] Wang Q. A study on the risks and influencing factors of catastrophic health expenditure of rural poor families: based on the 2018 CHARLS data. Chin J Health Policy. (2021) 14:44–9. doi: 10.3969/j.issn.1674-2982.2016.02.002

[34] Setoodehzadeh F Barfar E Ansari H Sari AA Azizi N. The economic burden of tuberculosis in Sistan: a high-risk region in Iran. Trop Med Int Health. (2021) 26:649–55. doi: 10.1111/tmi.13570, PMID: 33668078

[35] Liao T Xu L. Study on the influence of disease diagnosis level on the economic burden of pulmonary tuberculosis patients. Chin Health Serv Manag. (2009) 28:38–40. doi: 10.3969/j.issn.1003-0743.2009.01.012

[36] Bai L Xiao S Li Y Tang Y Gong D Tan Z. The impact of tuberculosis diagnosis-delay on the disease economic burden. Chin J Antitubere. (2012) 34:697–703. doi: 10.19982/j.issn.1000-6621.2012.11.003

[37] Chen S Zhang H Pan Y Long Q Xiang L Yao L. Are free anti-tuberculosis drugs enough? An empirical study from three cities in China. Infect Dis Poverty. (2015) 4:47. doi: 10.1186/s40249-015-0080-y, PMID: 26510711 PMC4625923

[38] Xu M Markström U Lyu J Xu L. Detection of low adherence in rural tuberculosis patients in China: application of Morisky medication adherence scale. Int J Environ Res Public Health. (2017) 14:248. doi: 10.3390/ijerph14030248, PMID: 28257075 PMC5369084

[39] Zhao L Liu J Hou W Cao S Lu Z. Study on the implementation status of cooperation of general hospitals and TB dispensaries strategy on tuberculosis control in China. Chin J Dis Control Prev. (2010) 14:1231–4.

